# Obliterated Brachial Artery, with Compound Fracture of the Humerus

**Published:** 1850-11

**Authors:** 


*Obliterated Brachial Artery, with Compound Fracture of the Humerus.*—When a limb has suffered compound fracture of a severe description, the question of amputation will be materially influenced by the amount of injury which the large vessels have sustained; and it has in general been found an excellent rule to remove the limb when an important artery is wounded, and the other circumstances of the case do not contra-indicate such an operation. But it may happen that a compound fracture may be accompanied by an injury to the principal artery of the limb, which, without wounding the same, may suspend the circulation through it; in such case the rule is not so clear, and the surgeon must evidently be guided by various considerations before he determines on a decided course. The more or less likelihood of the circulation being re-established by the collateral vessels must of course have considerable weight in the question. A case of this description came lately under the care of Mr. Hawkins, and we consider that it will be extremely useful to report it with full details, as such instances are calculated to serve as guides in cases of a similar nature. We are indebted for the particulars to Mr. Francis Day, one of Mr. Hawkins's dressers.

The patient is a market gardener, twenty-eight years of age, who was admitted into St. George's hospital, under the care of Mr. Hawkins, June 4th, 1850. He is tall and healthy in appearance, but of very drunken habits, and when received into the hospital, at three o'clock in the morning, he was in a state of intoxication and more or less collapse. An hour before, he had suffered a severe compound fracture of the right arm, caused by falling off the shafts of a wagon, the wheel of which passed over the arm. On examination, three wounds were seen near the seat of the fracture, the latter being situated about the upper part of the middle third of the humerus. One of the wounds was situated on the outer side of the arm; it was about two inches long, and bone was easily detected at the bottom of it, the soft parts within this solution of continuity being greatly destroyed. Two other wounds were noticed on the inner and the posterior part of the arm; muscle protruded through them, and blood likewise exuded, but to a small amount. The patient was reported not to have lost any more blood than was seen on the sleeve, which was saturated to some extent, but not alarmingly. The hand was perfectly cold, but as the whole surface of the body presented a very low temperature, the coldness of the hand was attributed partly to the state of collapse, and partly to the loss of blood.

The limb was placed on an angular splint, the edges of the wound brought together by a plaster, and three splints applied, one to the inner, a second to the outer, and the third to the anterior surface of the arm; the latter being raised on a cushion. The collapse having passed off in a few hours, the hand and forearm were found to be still cold; and sensation seemed to be more or less lost on the outer side of the thumb, and index finger, and at the back of the hand. Pulsation could be felt in the brachial artery, above the seat of the fracture, but none could be detected at the bend of the elbow, or at the radial or ulnar arteries. The hand had, however, retained its natural color, the face was flushed, and the patient seemed to be in great pain. A consultation was held in the middle of the day, when it was decided not to amputate the arm at once, but to wait and see whether collateral circulation would be established, reserving the removal of the limb till gangrene had commenced.

The patient, though he took an opiate, suffered great pain during the night, and the next day the forearm and hand were still cold, nor had any appreciable sensation returned to these parts. Ammonia, in saline draughts, and an opiate, were prescribed; but on the third day the skin at the end of the elbow was found in a state of gangrene, and vesicated; and as the wounds looked rather tense, a few incisions were made into them with great relief. Patient was ordered good diet and porter, but on the fourth day the gangrene began to spread on the outside of the arm, and severe symptomatic fever set in, with great pain in the side and abdomen. On the fifth day, the gangrene involved the deeper structures, and Mr. Hawkins then resolved, after consultation, to amputate immediately.

The patient was placed under the influence of chloroform, and Mr. Hawkins, taking his stand to the inside of the arm, so as to steady the comminuted parts during the process of sawing, removed the limb by the circular incision about an inch and a half from the shoulder-joint, the subclavian artery being compressed by Mr. Prescott Hewett above the clavicle.

The patient proceeded very favorably after the operation; nothing of importance happened during the next two months, at the expiration of which the stump was quite healed, and the patient discharged in good condition.

A careful dissection of the amputated arm showed that superficial gangrene had occurred in several places, affecting large patches of skin, both in the arm and forearm; in the latter, however, it was only in an incipient stage. Lymph and serum were effused in the subcutaneous tissues throughout the limb, but especially in the arm; in the latter region there were also large cavities containing ill-formed pus, both in the subcutaneous and deeper textures. The muscles at the middle part of the arm were much torn, and the bone, broken at its centre, was comminuted and bathed in pus. Both the superficial and deep veins were extensively clogged up by coagula, especially the venæ comites of the brachial artery. The latter was also found blocked up by a firm coagulum adhering to its internal coat, measuring about one inch in length. This coagulum was situated at about three inches above the elbow joint; the other portions of the artery were pervious, and quite sound, and the nerves were not found injured.

It would appear that the violence of the injury, when it reached the brachial artery, principally affected the inner and perhaps the middle coat, in the manner of the ligature, producing, like the latter, a complete obliteration of the vessel. It can hardly be wondered at that the collateral circulation did not become established, for that beautiful provision of nature may sometimes be tardy even in a perfectly sound limb, and partial gangrene take place, so that there was but a small chance of the collateral branches acting powerfully in this instance, as the whole limb had suffered so great a shock. Thus amputation was the only means of saving life, and the healing of the stump required no small amount of care, as the patient had undermined his health by habits of intemperance.—*London Lancet.*

